# P-2158. A Single Center's Utilization of Cell Free Microbial DNA Testing

**DOI:** 10.1093/ofid/ofae631.2312

**Published:** 2025-01-29

**Authors:** Alexander Shaffer, Erika Orner, Phyu Thwe, Liam S Conway-Pearson, Wendy Szymczak, Margaret E McCort

**Affiliations:** Montefiore Medical Center, Bronx, New York; Montefiore Medical Center, Bronx, New York; Montefiore Medical Center, Bronx, New York; Montefiore Medical Center, Bronx, New York; Montefiore Medical Center, Albert Einstein College of Medicine, Bronx, NY; Montefiore Medical Center / Albert Einstein College of Medicine, Bronx, New York

## Abstract

**Background:**

Metagenomic next generation sequencing techniques, either through targeted (tMGS) or shotgun sequencing (sMGS), have been shown to improve the odds of identifying a causative pathogen in a variety of disease states compared to conventional microbiologic testing. However, the high cost and unfamiliarity with MGS have led to questions on when and how these should best be used. We aimed to characterize the utilization of sMGS at a large urban academic hospital system, specifically the use of the commercial Karius test, a cell free microbial DNA (cfDNA) assay.

**Figure:**
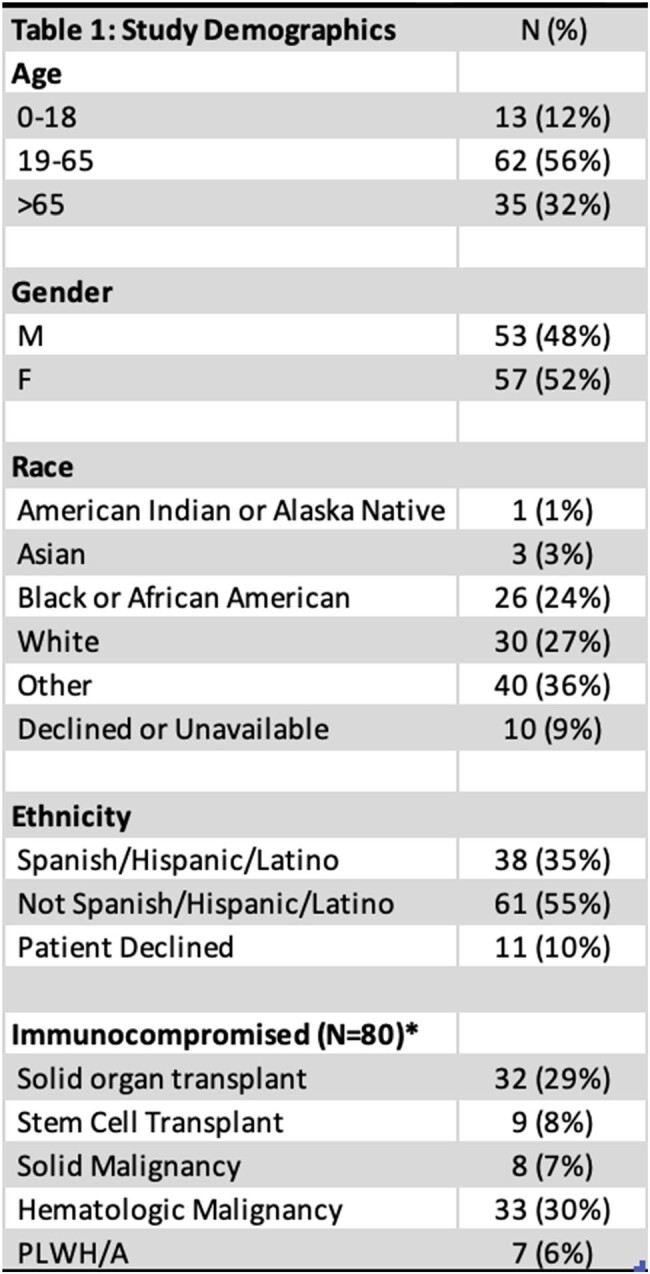

* conditions not mutually exclusive

**Methods:**

We conducted a retrospective observational study through detailed chart review of the Karius tests sent from our hospital system from June 13, 2020, through April 10, 2024. Charts were reviewed for demographic, clinical and outcome information. Statistical analysis was performed using chi-square analyses.

**Figure 1:**
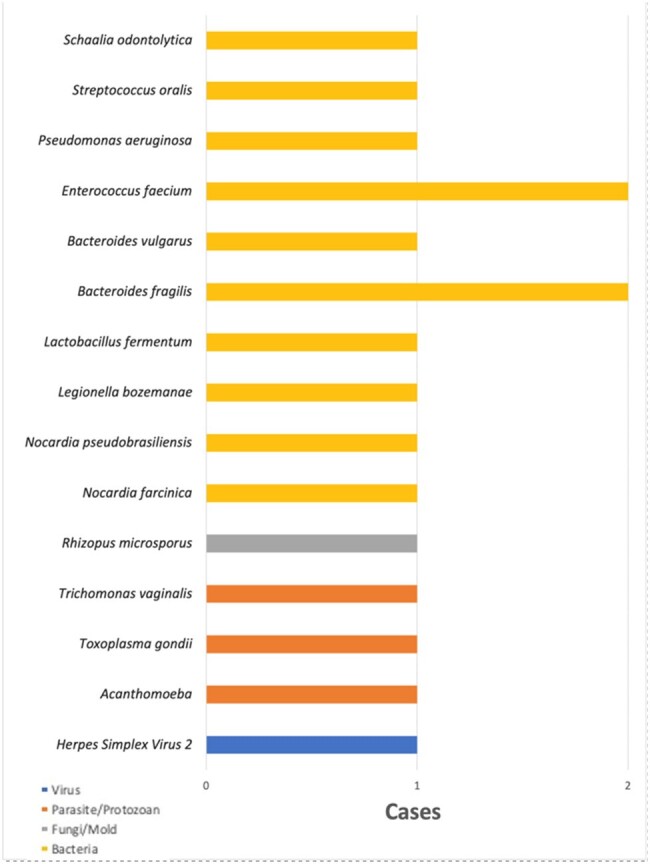
Pathogens with clinical additive diagnostic value (ADV)

**Results:**

110 Karius tests from 104 unique patients have been sent from our institution since 2020. Most patients were immunocompromised (72.7%); see Table 1 for demographics. Patients received on average 13.4 days of antimicrobials before cfDNA testing was sent. An average of 6.5 blood cultures were sent, and in 69 cases, invasive procedures were performed before Karius testing was sought.

A pathogen was detected by Karius in 62 cases (56%). Results led to a change in antimicrobial management in 23% of cases. Karius had clinical additive diagnostic value (ADV) in 17 cases (15.6%), as defined by the detection of a causative pathogen that was not detected on conventional testing. Pathogens with clinical ADV are shown in Figure 1. There was no statistically significant difference between ADV, pathogen detection, or test concordance with conventional testing (p=0.71, p=0.64, and p=0.50 respectively) for cfDNA use in immunocompetent versus immunocompromised patients.

**Conclusion:**

sMGS testing can provide clinically impactful results, although the optimal patient population and clinical scenario in which to employ these tests remains undetermined. Despite significant antimicrobial exposure prior to cfDNA testing, the Karius assay detected several fastidious pathogens that may not have been detected by conventional methods. Further research into ideal diagnostic utilization and stewardship of these tools is needed.

**Disclosures:**

Wendy Szymczak, PhD, Quidel: Advisor/Consultant

